# miR-181a regulates the host immune response against *Schistosoma japonicum* infection through the TLR4 receptor pathway

**DOI:** 10.1186/s13071-021-05063-z

**Published:** 2021-10-24

**Authors:** Yixiao Tang, Yuanxi Shen, Yang Hong, Zuhang Zhang, Qi Zhai, Zhiqiang Fu, Hao Li, Ke Lu, Jiaojiao Lin

**Affiliations:** 1 National Reference Laboratory for Animal Schistosomiasis, Shanghai Veterinary Research Institute, Chinese Academy of Agricultural Sciences, Shanghai, 200241 P.R. China; 2 Key Laboratory of Animal Parasitology of Ministry of Agriculture, Shanghai Veterinary Research Institute, Chinese Academy of Agricultural Sciences, Shanghai, 200241 P.R. China

**Keywords:** *Schistosoma japonicum*, miR-181a, *Microtus fortis*, TLR4 receptor pathway, Cytokines

## Abstract

**Background:**

Schistosomiasis japonica is a serious zoonotic parasitic disease. Preliminary studies have shown that the expression of microRNA-181a (miR-181a) in the liver, lung and spleen tissues of susceptible host BALB/c mice and resistant host reed vole (*Microtus fortis*) 10 days post-infection (dpi) with *Schistosoma japonicum* was significantly different from pre-infection levels. This difference suggests the possibility that miR-181a expression may be related to the regulation of the hosts’ early immune response against *S. japonicum* infection and thereby affect the development and survival of parasites in their final hosts.

**Methods:**

BALB/c mice, *M. fortis*, Toll-like receptor 4 (TLR4)-deficient mice and wild-type mice (C57BL/6) were infected with *S. japonicum*, and differences in miR-181a expression between BALB/c mice and *M. fortis* over different time points post-infection (0, 3, 7, 10 and 14 dpi) were compared. MiR-181a mimic, miR-181a inhibitor and irrelevant miRNA, as well as lipopolysaccharide (LPS), a TLR4 receptor ligand, were used to transfect mouse RAW264.7 macrophages. The expression levels of the TLR4 pathway-related cytokines interleukin (IL)-1β, tumor necrosis factor α (TNF-α) and IL-6 were detected by quantitative PCR analysis.

**Results:**

The expression of miR-181a was significantly upregulated in the serum and liver of mice infected with *S. japonicum* and downregulated in the serum and liver of *M. fortis*. T-helper cell (Th1)-type cytokines, such as TNF-α, IL-6 and IL-1β, and Th2-type cytokines, such as IL-10 and IL-4, were differentially expressed in *M. fortis* and BALB/c mice in the early stage of infection. The expression level of miR-181a in the serum was threefold higher in TLR4-deficient mice than in wild-type mice 10 dpi with *S. japonicum*. The expression of IL-1β, TNF-α and IL-6 decreased in RAW264.7 cells transfected with miR-181a mimic and increased in cells transfected with miR-181a inhibitor. miR-181a expression was downregulated and the expressions of TLR4 and three TLR4 pathway-related cytokines (IL-1β, IL-6, and TNF-α) were upregulated in RAW264.7 macrophages stimulated with the TLR4 receptor ligand LPS.

**Conclusion:**

These results suggest the possibility of mutual regulation between miR-181a and the TLR4 signaling pathway during *S. japonicum* infection. miR-181a may regulate the expression of pro-inflammatory factors through the TLR4 receptor pathway and participate in the immunomodulatory effect of anti-*S. japonicum* infection.

**Graphical abstract:**

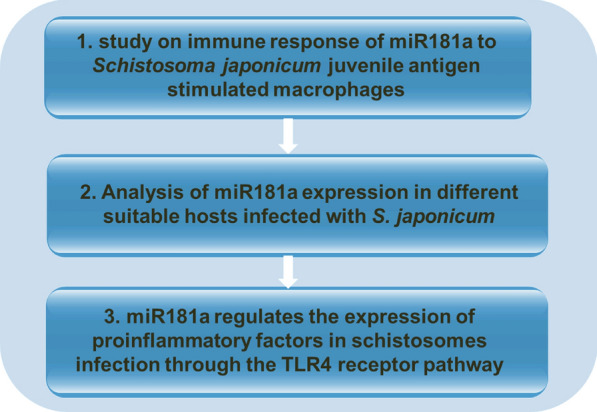

## Background

Schistosomiasis is a tropical and subtropical parasitic disease caused by schistosome infection, with an estimated 200 million people infected and 800 million people living in schistosomiasis-endemic regions. Studies have shown that during the early period of schistosome infection in mice, the induction of a T-helper cell type I (Th1)-dominated immune response against schistosome infection is characterized by increased levels of interferon-γ (IFN-γ) and tumor necrosis factor α (TNF-α), and that the host immune response shifts to a Th2-dominant immune response 4–6 weeks after infection which is characterized by increased expression levels of several interleukins (ILs), including IL-4, IL-13 and IL-12 [1]. Increased expression of these Th1- and Th2-associated cytokines is important in terms of host resistance to schistosome infection. Macrophages play a vital role in host resistance to schistosome infection and pathological changes in the liver. Macrophages can be activated into M1 macrophages and M2 macrophages. M1 macrophages are activated by the secretion of IFN-γ (mainly secreted by TH1 cells, cytotoxic T cells and natural killer [NK] cells), lipopolysaccharide (LPS; a component of the outer membrane of Gram-negative bacteria) and granulocyte–macrophage colony-stimulating factor (GM-CSF) and mainly produce pro-inflammatory cytokines [2–4], such as TNF-α, IL-1β and IL-12. M2 macrophages are activated by Th2 cytokines and then play a regulatory role to maintain this chronic inflammatory disease. In contrast to M1 macrophages, M2 macrophages are involved in the Th2 bias response, producing transforming growth factor β (TGF-β), IL-10, IL-13 and IL-4, which leads to parasite clearance and host protection [5–7].

 The reed vole *Microtus fortis* is the only mammal host identified to date that exhibits resistance against schistosome infection. Studies have shown that *M. fortis* has a certain resistance to *S. japonicum* infection [8–11]. Such resistance can affect the growth and development of *S. japonicum* in the host, resulting in immature development of the schistosomes and the female not laying eggs, thereby protecting the host from severe disease [12]. Preliminary studies in our laboratory have also shown that 10 days after the non-susceptible host *M. fortis* and the susceptible host mice were infected with *S. japonicum*, expression of microRNA-181a (miR-181a) in liver, lung and spleen tissues was significantly different, possibly related to the regulation of host resistance to schistosome infection. An in-depth analysis of the differences in the expression of miR-181a and the effect of this differential expression of related cytokines in *M. fortis* and mice infected with schistosomes is highly relevant for elucidating the immune regulation mechanism of miR-181a molecules in schistosome infection and the interaction mechanism between hosts and *S. japonicum*. In the present study, we collected liver tissue and serum samples from C57BL/6 mice and *M. fortis* infected with *S. japonicum* cercariae at different post-infection periods, and then analyzed and compared the expression levels of miR-181a in these two different suitable hosts after infection with *S. japonicum*. Our ultimate aim was to provide a basis for further understanding of the regulatory role of host miR-181a in the growth and development of schistosomes.

Toll-like receptors (TLRs) are a class of pathogenic pattern receptors that mediate innate immunity and are mainly involved in the recognition of molecular patterns associated with pathogenic microorganisms. It has been shown that transcription factors of signaling molecules can play a key role in regulating microRNA (miRNA) expression in the TLR signaling pathway by acting as transcriptional activators of miRNAs, which in turn initiate miRNA expression. Also, miRNAs have complex and subtle regulatory effects on the TLR signaling pathway, and inhibition of the TLR signaling pathway is crucial in suppressing excessive inflammatory responses [13]. These two processes regulate each other and interact with each other, thus forming a complex regulatory network.

TLR4 was first identified in 1997 with elevated expression in the inflammatory response [14]. It was the first TLR-related protein to be identified in humans and was subsequently found to be distributed in almost all cell lines, but mostly expressed in cells involved in host immune and defense functions, such as monocytes, lymphocytes, dendritic cells, granulocytes, epithelial cells, as well as more recently in renal tubular epithelial cells, heart respiratory epithelial cells and intestinal epithelial cells [15, 16]. TLR4 can recognize and transmit signals by recognizing specific antigenic components of pathogen-associated molecular patterns. LPS is an important TLR4 ligand [17], and in response to LPS stimulation, it causes impaired activation of downstream cytokines [18]. Studies on humans have shown that LPS-mediated cytokine release is reduced in surgical patients, indicating a reduction in the innate immunity of cells to inflammation, which is not conducive to the recovery of the body [19]. The TLR4 signaling pathway is one of the more important inflammatory pathways as well as a relatively well-studied one, and this pathway is closely associated with the occurrence and development of many diseases. The expression of downstream inflammatory factors associated with TLR4 often affects the developmental direction of the disease and the post-cure situation. The relationship between the TLR pathway and miRNAs is crucial in terms of the maintenance of normal physiological functions in the body and the prevention of excessive inflammation and disease development. Moreover, study of this relationship provides a strategy for gaining a better understanding of diseases associated with the TLR signaling pathway. More specifically, exploring the relationship between the two is vital for furthering our understanding of host resistance to schistosome infection.

## Methods

### Schistosome infection and sample preparation

Six-week-old male BALB/c mice were purchased from Shanghai Slac Laboratory Animal Co., Ltd. (Shanghai, P.R. China) and the reed voles (*M. fortis*) (each weighing about 60 g) were kindly provided by the Shanghai Experimental Animal Center (Shanghai, P.R. China). Ten BALB/c mice and ten *M. fortis* were each infected with 40 *S. japonicum* cercariae via the abdomen. Blood samples were taken from the orbit on 0, 3, 7, 10 and 14 days post-infection (dpi). Subsequently, serum and liver samples were collected, and miRNA was extracted to measure the expression of miR-181a in mice and *M. fortis* at different time periods.

TLR4-deficient mice and C57BL/6 wild-type mice (WT; control group) were purchased from Nanjing Biomedical Research Institute of Nanjing University (Gulou, Nanjing, P.R. China). Five TLR4-deficient mice and five WT mice were infected with 40 *S. japonicum* cercariae via the abdominal skin patch method, and serum was collected by dissection at 10 dpi and stored at − 80 °C for later use. The *S. japonicum* cercariae were provided by Shanghai Veterinary Research Institute, Chinese Academy of Agricultural Sciences (Shanghai, P.R. China).

### Culture and stimulation of RAW264.7 macrophages

RAW264.7 macrophages were purchased from Applied Biological Materials Inc. (Richmond, BC, Canada), maintained at the Shanghai Veterinary Research Institute, Chinese Academy of Agricultural Sciences (Shanghai, P.R. China) and cultured in Dulbecco’s Modified Eagle’s Medium (DMEM; Invitrogen, Thermo Fisher Scientific, Waltham, MA, USA) supplemented with 10% heat-inactivated fetal bovine serum (FBS; Invitrogen, Thermo Fisher Scientific) at 37 °C, 5% CO_2_ in an incubator. Cells were cultured into a 12-well culture plate at a density of 2 × 10^5^ cells/well. When the cells were spread over 70% of the bottom, they were first stimulated with 20 µg/ml LPS for 12 h, following which 20 nM miR-181a mimic, miR-181a inhibitor or irrelevant miRNA control (GenePharma, Shanghai, P.R. China) was added to a well. The cells were then transfected with Lipofectamine 2000 transfection reagent (Life Technologies, Thermo Fisher Scientific) according to the manufacturer’s instructions for 4 h and then collected for further study.

### Extraction of RNA and miRNA and detection of cytokines and miR-181a

The TRIzol reagent (Invitrogen, Thermo Fisher Scientific) method was used to extract total RNA of cells, serum and liver tissue according to the manufacturer’s instructions. The Tiangen kit (TIANGEN Biotech Co., Beijing, P.R. China) was used to extract miRNA, and the SYBR Green Master Mix kit (Applied Biosystems, Thermo Fisher Scientific, Waltham, MA, USA) was used to determine the expression levels of miR-181a and cytokines TNF-α, IL-4, IL-6, IL-10 and IL-1β (Table 1).

**Table1:** Primer names and primer sequence of quantitative PCR

Primer names	Primer sequence
IL-4-F	GGTCTCAACCCCCAGCTAGT
IL-4-R	GCCGATGATCTCTCTCAAGTGAT
IL-6-F	CTGCAAGAGACTTCCATCCAG
IL-6-R	AGTGGTATAGACAGGTCTGTTGG
IL-10-F	CTTACTGACTGGCATGAGGATCA
IL-10-R	GCAGCTCTAGGAGCATGTGG
TNF-α-F	CCTGTAGCCCACGTCGTAG
TNF-α-R	GGGAGTAGACAAGGTACAACCC
GAPDH-F	AGGTCGGTGTGAACGGATTTG
GAPDH-R	TGTAGACCATGTAGTTGAGGTCA
U6-F	CTCGCTTCGGCAGCACA
U6-R	AACGCTTCACGAATTTGCGT

F, Forward; GAPDH, glyceraldehyde 3-phosphate dehydrogenase; R, reverse

The cells, serum and liver tissue of mice and *M. fortis* were transferred into Eppendorf tubes (Eppendorf AG, Hamburg, Germany). Lysate and miRNA Homogonote Addictive were added to the tubes and the contents mixed. Then, 300 µl of acidic phenol was added to the tubes, and the contents were mixed to achieve separation. The liquid phase was transferred into a new tube, followed by the addition of anhydrous ethanol and mixing. Finally, miRNA wash solution was added into the filter column. After centrifugation, the contentration of miRNA was measured.

The PCR thermocycling program consisted of one cycle at 50 °C for 2 min, one cycle at 90 ℃ for 15 s, followed by 40 cycles of 95 °C for 5 s and 60 °C for 34 s, with the end time point of 60 °C (34 s) being the fluorescence signal detection point; each reaction was repeated three times. The quantitative PCR reaction (qPCR) was carried out according to the parameters specified in the Tiangen Extraction Kit (DP501; TIANGEN Biotech Co.); Applied Biosystems 7500 Real-Time PCR Software v2.0.5 was used for all calculations and analyses; GAPDH and U6 were used as the internal references for messenger RNA (mRNA) and miRNA quantification, respectively; and the relative expression of target genes was calculated by the 2^−ΔΔ^Ct method.

### Statistical analysis

The experimental data were compared using SPSS version 22.0 statistical software (SPSS IBM Corp., Armonk, NY, USA). The t-test was used for two independent samples, and one-way analysis of variance and the least significant difference test was used for multiple groups. Significance was set at *P* < 0.05, with *P* < 0.01 indicating an extremely significant difference. All experiments were repeated a minimum of three separate times. GraphPad Prism 5 (GraphPad Software Inc., San Diego, CA, USA) was used for graphing the results.

## Results

### Analysis of miR-181a expression in the serum of mice and *M. fortis* infected with *S. japonicum*

**Fig. 1:**
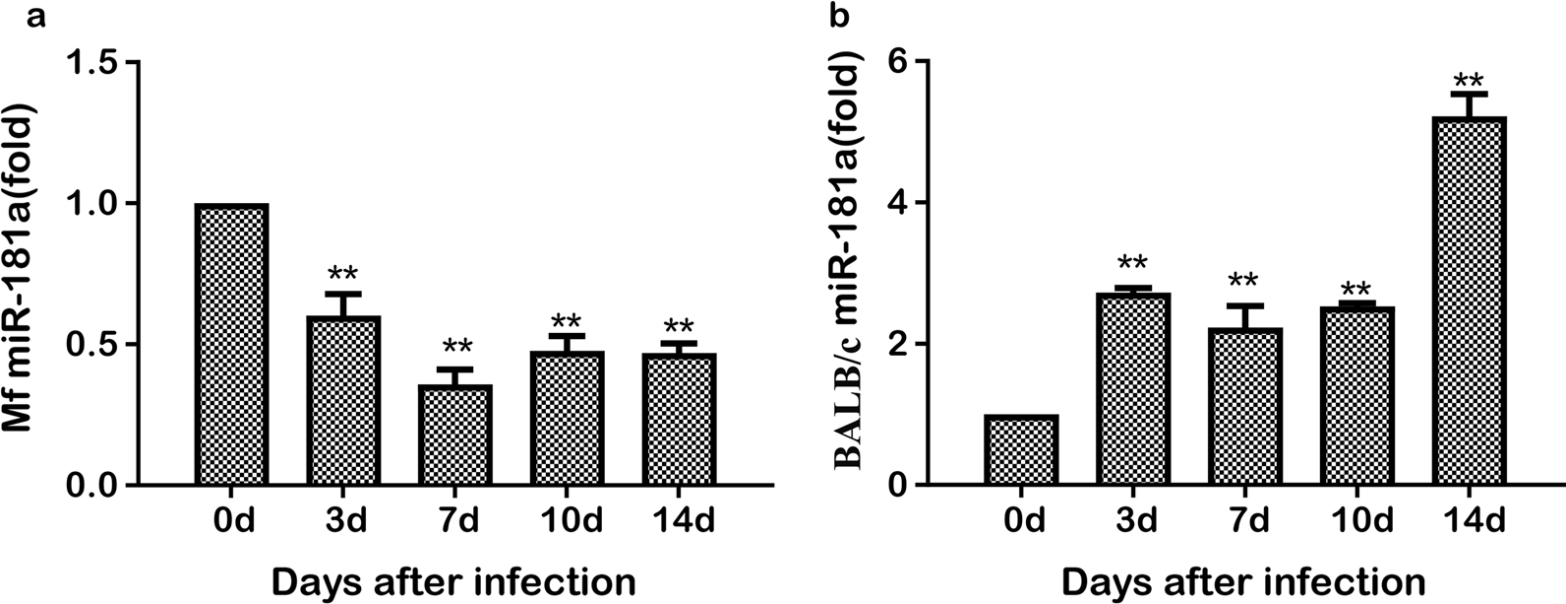
Analysis of the expression level of miR-181a in the serum of male BALB/c mice (**a**) and reed vole *Microtus fortis* (**b**) infected with *Schistosoma japonicum* at different time periods post-infection. Asterisks indicate significant difference at *P < 0.05, **P < 0.01. Abbreviations: MF, *Microtus fortis*

As shown in Fig. 1, the expression of miR-181a in the serum of *M. fortis* infected with *S. japonicum* at 3, 7, 10 and 14 dpi, respectively was significantly lower than that in the uninfected control group at the same time points (Fig. 1a). In comparison, the expression level of miR-181a in mice infected with *S. japonicum* kept increasing over time and was significantly higher at 3, 7, 10 and 14 dpi, respectively, than that in the uninfected control group at the same time points (Fig. 1b).

### Analysis of miR-181a expression in the liver of mice and *M. fortis* infected with *S. japonicum*

**Fig. 2:**
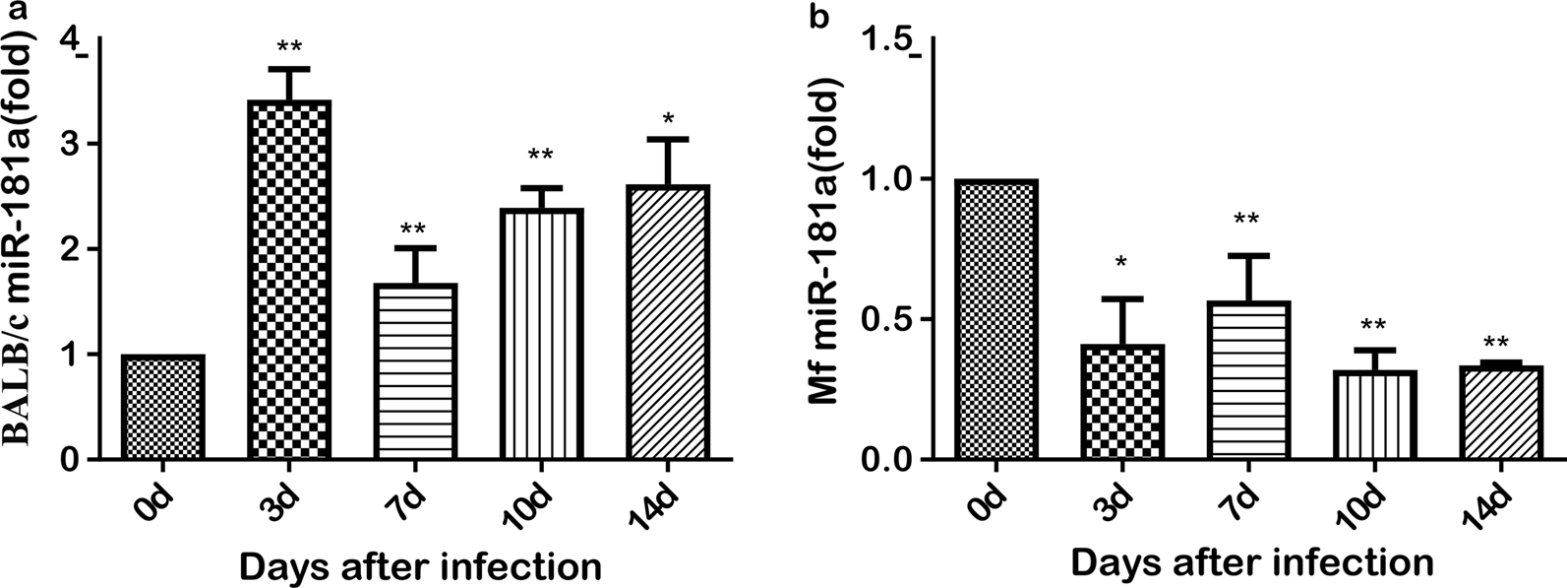
Analysis of the expression level of miR-181a in the livers of male BALB/c mice (**a**) and *M. fortis* (**b**) infected with *S. japonicum* at different time points post-infection. Asterisks indicate significant difference at *P < 0.05, **P < 0.01

The livers of mice and *M. fortis* were collected separately, and total RNA was extracted to determine the changes in miR-181a expression. The results showed that the expression level of miR-181a in the livers of schistosome-infected mice at 3, 7, 10 and 14 dpi, respectively, was significantly higher than that in the uninfected control group at the same time points (Fig. 2a), while the expression level of miR-181a in the livers of schistosome-infected *M. fortis* at 3, 7, 10 and 14 dpi, respectively, was significantly lower than that of the uninfected control group (Fig. 2b).

### Analysis of changes in cytokine levels in mice and* M. fortis* at the early stage of schistosome infection

**Fig. 3:**
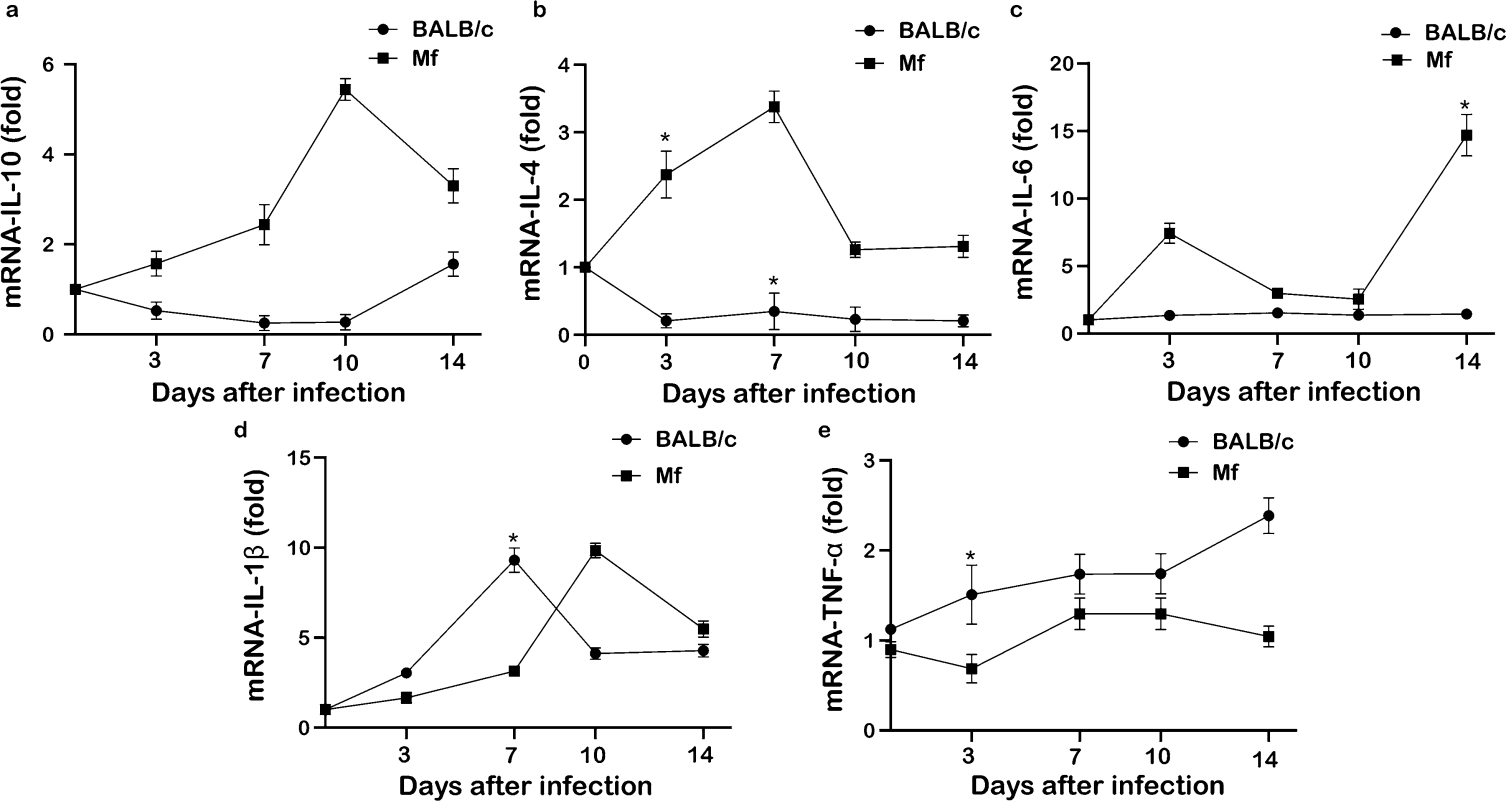
Changes in the expression of IL-10 (**a**), IL-4 (**b**), IL-6 (**c**), IL-1β (**d**), and TNF-α (**e**) in mice and *M. fortis* after schistosome infection. Asterisk indicates significant difference at *P < 0.05

Changes in the expression of cytokines IL-10, IL-4, IL-6, IL-1β and TNF-α were measured in BALB/c mice, the susceptible host of *S. japonicum*, and in *M. fortis*, the resistant host, before infection and on 3, 7, 10 and 14 dpi, respectively. The results showed that the expression levels of anti-inflammatory factors IL-10 (Fig. 3a), IL-4 (Fig. 3b) and IL-6 (Fig. 3c) were higher in *M. fortis* than in mice and that the expression level of TNF-α (Fig. 3e) was lower in *M. fortis* than in mice. The expression level of IL-1β (Fig. 3d) was higher in mice than in *M. fortis* on 3 and 7 dpi and lower than that in *M. fortis* on 10 and 14 dpi. During the early stage of infection, the expression of the five detected cytokines, with the exception of IL-4, was generally upregulated post-schistosome infection in *M. fortis*. In mice, IL-10 and IL-4 expression was downregulated, IL-6 expression was not significantly changed and TNF-α expression was upregulated and IL-1β expression was upregulated on 3 and 7 dpi and downregulated on 10 and 14 dpi (see Fig. 3).

### Expression of miR-181a in TLR4-deficient mice

**Fig. 4:**
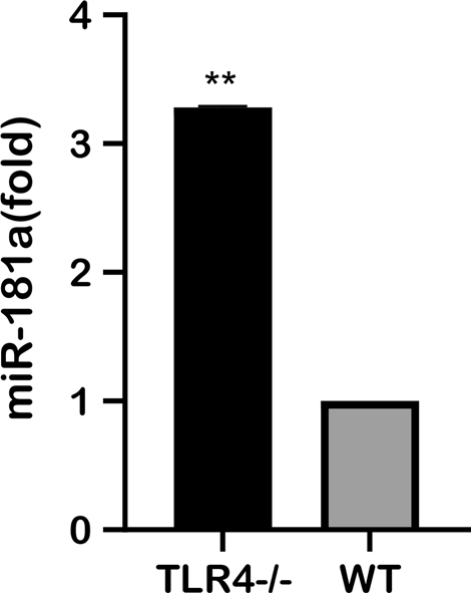
Analysis of miR-181a in serum of TLR4-deficient mice and C57BL/6 mice 10 days after infection with *S. japonicum*. Asterisk indicates significant difference at **P < 0.01. Abbreviation: TLR4, Toll-like receptor 4; WT wild type

TLR4-deficient mice and C57BL/6 mice (WT mice) were respectively infected with *S. japonicum* cercariae. Serum was collected 10 days later and the level of miR-181a was measured. The results showed that the level of miR-181a was significantly higher (3.8-fold) in TLR4-deficient mice than in WT mice and that the difference was highly significant (Fig. 4).

### Analysis of miR-181a and TLR4 expression after LPS stimulation of macrophages

**Fig. 5:**
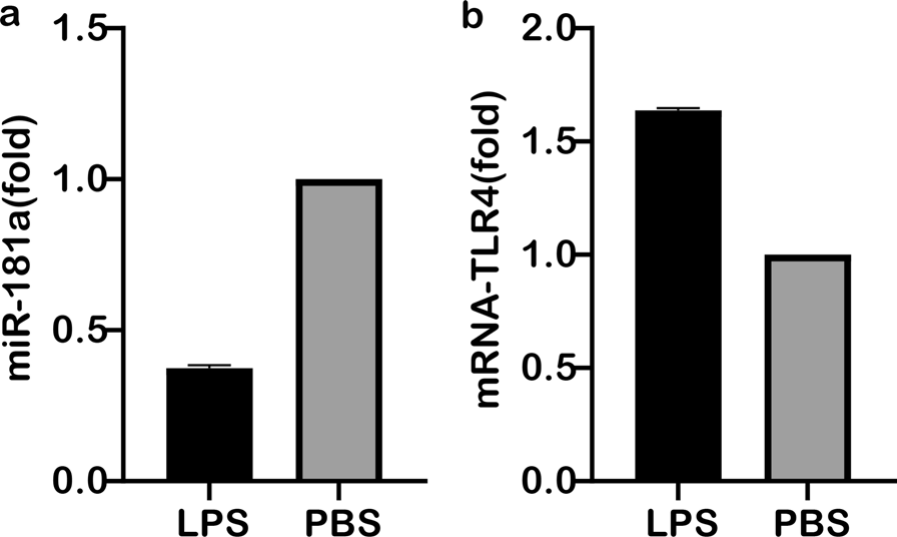
Expression analysis of miR-181a (**a**) and TLR4 (**b**) after LPS stimulation of RAW264.7 macrophages. Abbreviation: PBS, Phosphate buffered saline

RAW264.7 macrophages were stimulated with LPS. Compared with the untreated blank control group, the expression of miR-181a was significantly downregulated in RAW264.7 macrophages after 12 h of LPS stimulation (about 40% of that of the control group) (Fig. 5a). The difference was very significant. In comparison, expression of TLR4 was upregulated to about 1.5-fold that of the control group; this difference was also very significant, as shown in Fig. 5b.

### Cytokine expression after LPS stimulation of RAW264.7 cells

**Fig. 6:**
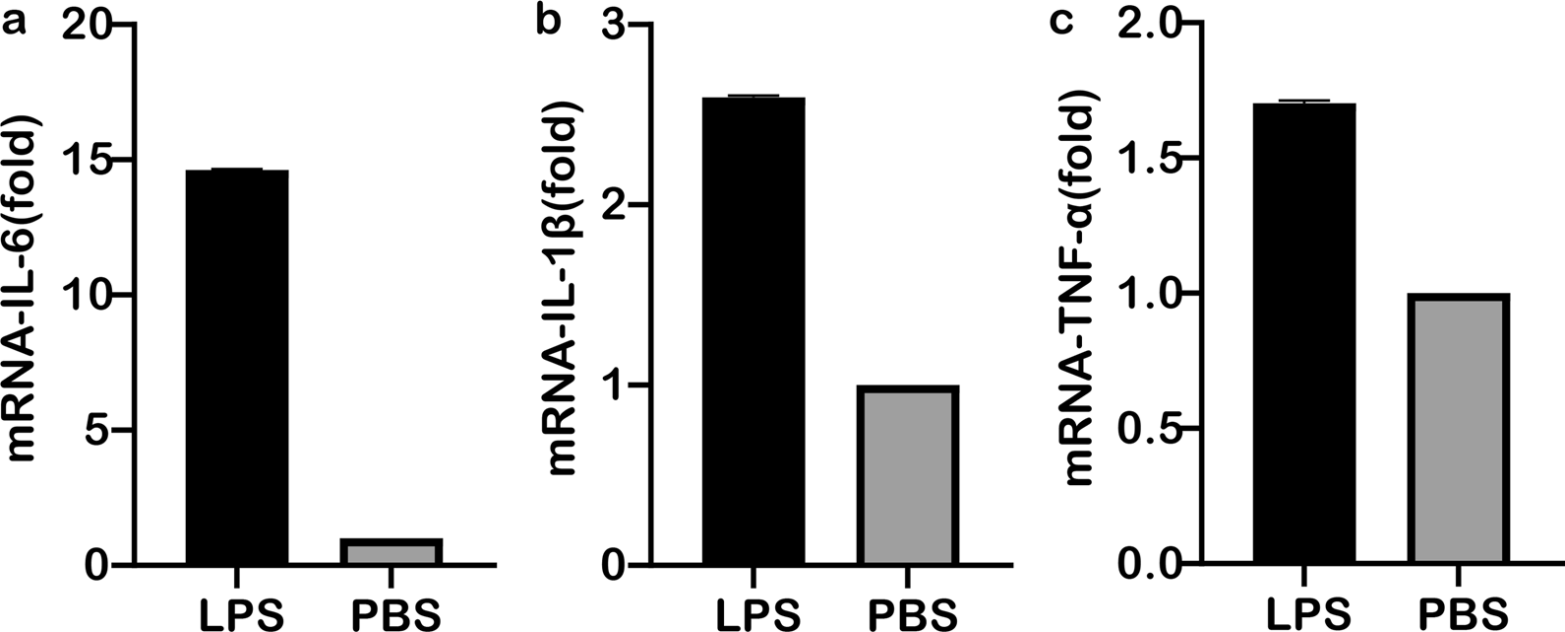
The expression of IL-6 (**a**), IL-1β (**b**), TNF-α (**c**) after LPS stimulation of RAW264.7 macrophages

Following the stimulation of RAW264.7 cells with LPS, a TLR4 receptor ligand, the expression levels of IL-6, IL-1B and TNF-α in the cells were detected by qPCR. The results showed that the expression levels of IL-6 (Fig. 6a), IL-1β (Fig. 6b) and TNF-α (Fig. 6c) were significantly higher in RAW264.7 cells treated with LPS (by approx. 12.5-, 2.8- and 1.6-fold, respectively) than in the unstimulated control group and that the differences were all very significant.

### Analysis of miR-181a regulation on the expression of TLR4 receptor pathway inflammatory factors

**Fig. 7:**
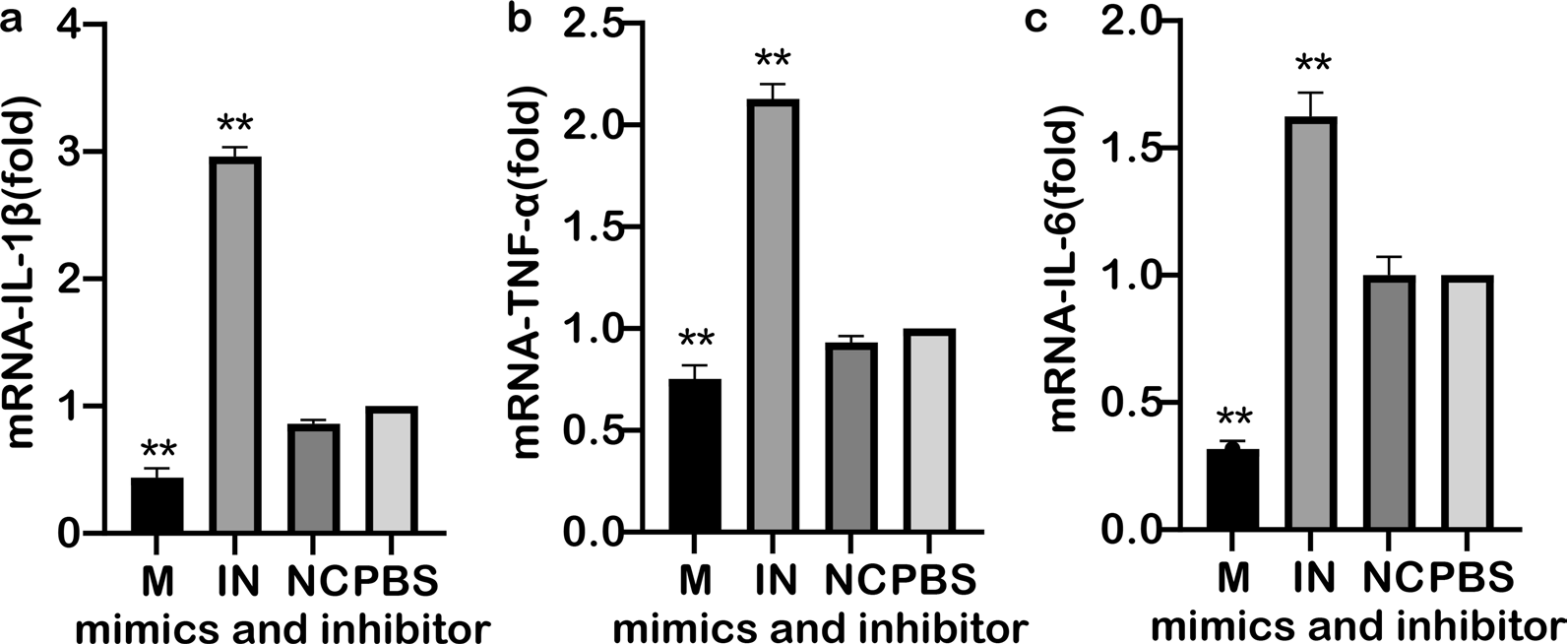
Analysis of the expression of IL-1β (**a**), TNF-α (**b**) and IL-6 (**c**) after transfection of RAW264.7 cells with miR-181a mimic and miR-181a inhibitor. Asterisks indicate significant different at **P < 0.01. Abbreviations: IN, Inhibitor; M, miR-181a mimic

RAW264.7 cells were transfected with miR-181a mimic, miR-181a inhibitor and irrelevant microRNA, respectively, and then changes in the expression of cytokines IL-1β (Fig. 7a), TNF-α (Fig. 7b) and IL-6 (Fig. 7c) in the cells were detected. Comparison of the results with the non-transfection phosphate buffered saline control group showed that the expression of all three cytokines decreased by varying degrees after transfection with miR-181a mimic and increased significantly in cells transfected with miR-181a inhibitor; in contrast, transfection with irrelevant control miRNA resulted in no significant difference in expression level (Fig. 7). These results suggest that miR-181a may play a negative regulatory role on the expression of these three Th1-type cytokines in RAW264.7 cells.

## Discussion

Following the infection of *M. fortis* with *S. japonicum*, the development of schistosomes became stunted and abnormal, with most dying within 2 weeks after infection and only very few surviving until the third week post-infection. In this study, we used real-time qPCR technology to further analyze the difference in miR-181a expression in the serum and liver of mice and *M. fortis* at different post-infection time points. The results showed that mir-181a expression was significantly upregulated and downregulated in the serum and liver of the schistosomiasis-infected mice and *M. fortis* respectively, at the different post-infection time points. Our recent study also showed that miR-181a negatively regulates the immune response of the schistosomulum antigen of *S. japonicum*, stimulates macrophages and promotes the conversion of macrophages to M2-type cells [20]. In the present study, we compared the expression levels of the Th1-type cytokines IL-1β, TNF-α and IL-6 and the Th2-type cytokines IL-4 and IL-10 in susceptible host BALB/c mice and resistant host *M. fortis* on different days post-infection with *S. japonicum*. The results showed that during the early period of infection, the expression levels of the two Th2-type cytokines IL-10 and IL-4 were higher in *M. fortis* than in the BALB/c mice. Among the three Th1-type cytokines detected, the expression level of IL-6 was higher in *M. fortis* than in BALB/c mice, the expression level of TNF-α was lower in *M. fortis* than in BALB/c mice, and the expression level of IL-1β was lower in *M. fortis *than in BALB/c mice on 3 and 7 dpi and higher in *M. fortis* than BALB/c mice on 10 and 14 dpi. At the same time, the expression of all five detected cytokines was generally upregulated at all post-inoculation measurement points in *M. fortis* compared with pre-infection levels. The expression of the two Th2-type cytokines IL-10 and IL-4 in mice was downregulated during the first 10 days post-infection. Among the three detected Th1 cytokines, the expression level of IL-6 was not significantly changed, TNF-α was upregulated and IL-1β was upregulated on 3 and 7 dpi, and downregulated on 10 and 14 dpi. Taken together, these results further suggested that miR-181a played a key role in the early immune regulation of host resistance to schistosome infection. After the *S. japonicum*-susceptible host, namely the mice, were infected with schistosomes, upregulated expression of miR-181a may negatively regulate the host immunity against schistosome infection by downregulating the expression of Th2-type cytokines IL-10, IL-4, among others. In contrast, the downregulated expression of miR-181a in the resistant host, namely *M. fortis*, may enhance the host immunity against schistosome infection by upregulating the expression of Th2-type cytokines IL-10 and Th1-type cytokines IL-1β, TNF-α and IL-6. These may be some of the factors which affect the survival and development of schistosomes in these two hosts showing different susceptibility.

TLR4 is a crucial pathway which activates the NF-kB signaling pathway and plays an important role in the immune response of the host [21]. Research by Han Hongxiao and his colleagues revealed that some differential expression miRNAs between *S. japonicum*-susceptible host mice and the resistant host *M. fortis* may be related to the regulation of the TLR signaling pathway [22]. LPS is considered to be a bacterial endotoxin that stimulates the immune response of the host and acts mainly through activation of the TLR4 receptor pathway [23]. To understand the immunomodulatory mechanism of miR-181a, in the present study we transfected RAW264.7 cells with miR-181a mimic and inhibitor, respectively. The results showed that compared with the non-transfection control cells, the expression of three TLR4 pathway-related inflammatory factors, including IL-1β, TNF-α and IL-6, decreased to varying degrees after transfection with miR-181a mimic, while the expression of these three cytokines was significantly increased in cells transfected with miR-181a inhibitor, indicating that miR-181a negatively regulated the expression of these three TLR4 pathway-related cytokines in RAW264.7 cells. The finding that the level of miR-181a in serum was significantly higher in *S. japonicum*-infected TLR4-deficient mice than in WT mice further verified the correlation between miR-181a and the TLR4 pathway. After stimulation of RAW264.7 macrophages with LPS, a TLR4 receptor ligand, the expression of miR-181a in the cells was significantly downregulated and the expression of TLR4 was upregulated. At the same time, the resultant expression of three TLR4 pathway-related cytokines, IL-1β, TNF-α and IL-6, was significantly upregulated. These results suggested that there may be complex and subtle interactions between miR-181a and the TLR4 signaling pathway in *S. japonicum* infection. MiR-181a may regulate the expression of pro-inflammatory factors through the TLR4 receptor pathway and participate in the immune response against schistosome infection.

## Conclusions

There may be mutual regulation between miR-181a and the TLR4 signaling pathway during *S. japonicum* infection. MiR-181a may regulate the expression of cytokines and other factors through the TLR4 receptor pathway and thus participate in the immunomodulatory effect of anti-*S. japonicum* infection.

## Data Availability

The datasets used or analyzed during the current study are available from the corresponding author on reasonable request.

## Abbreviations

DMEM: Dulbecco’s Modified Eagle’s Medium
FBS: Fetal bovine serum
GM-CSF: Granulocyte-macrophage colony-stimulating factor
IFN-γ: Interferon-γ
IL: Interleukin
LPS: Lipopolysaccharide
miR-181a: MicroRNA-181a
miRNA: MicroRNA
TGF-β: Transforming growth factor β
Th1: T-helper cell type 1
TNF-α: Tumor necrosis factor α
